# Characterization of age‐associated exhausted CD8^+^ T cells defined by increased expression of Tim‐3 and PD‐1

**DOI:** 10.1111/acel.12435

**Published:** 2016-01-10

**Authors:** Kyoo‐A Lee, Kwang‐Soo Shin, Ga‐Young Kim, You Chan Song, Eun‐Ah Bae, Il‐Kyu Kim, Choong‐Hyun Koh, Chang‐Yuil Kang

**Affiliations:** ^1^ Laboratory of ImmunologyResearch Institute of Pharmaceutical SciencesCollege of PharmacySeoul National University1 Gwanak‐ro, Gwanak‐guSeoul151‐742Korea; ^2^ Department of Molecular Medicine and Biopharmaceutical ScienceGraduate School of Convergence Science and TechnologySeoul National University1 Gwanak‐ro, Gwanak‐guSeoul151‐742Korea

**Keywords:** Aging, CD8^+^ T cells, T‐cell exhaustion, Tim‐3, PD‐1

## Abstract

Aging is accompanied by altered T‐cell responses that result in susceptibility to various diseases. Previous findings on the increased expression of inhibitory receptors, such as programmed cell death protein 1 (PD‐1), in the T cells of aged mice emphasize the importance of investigations into the relationship between T‐cell exhaustion and aging‐associated immune dysfunction. In this study, we demonstrate that T‐cell immunoglobulin mucin domain‐3 (Tim‐3), another exhaustion marker, is up‐regulated on aged T cells, especially CD8^+^ T cells. Tim‐3‐expressing cells also produced PD‐1, but Tim‐3^+^ PD‐1^+^ CD8^+^ T cells had a distinct phenotype that included the expression of CD44 and CD62L, from Tim‐3^−^ PD‐1^+^ cells. Tim‐3^+^ PD‐1^+^ CD8^+^ T cells showed more evident properties associated with exhaustion than Tim‐3^−^ PD‐1^+^ CD8^+^ T cells: an exhaustion‐related marker expression profile, proliferative defects following homeostatic or TCR stimulation, and altered production of cytokines. Interestingly, these cells produced a high level of IL‐10 and induced normal CD8^+^ T cells to produce IL‐10, which might contribute to immune dysregulation in aged mice. The generation of Tim‐3‐expressing CD8^+^ T cells in aged mice seems to be mediated by encounters with antigens but not by specific infection, based on their high expression of CD49d and their unbiased TCR Vβ usage. In conclusion, we found that a CD8^+^ T‐cell population with age‐associated exhaustion was distinguishable by its expression of Tim‐3. These results provide clues for understanding the alterations that occur in T‐cell populations with age and for improving dysfunctions related to the aging of the immune system.

## Introduction

Aging is accompanied by an increase in susceptibility to various infections due to altered immune responses (Gavazzi & Krause, 2002). In particular, multiple changes in T‐cell populations (e.g., impaired thymic output, decreased naïve T cells and increased memory phenotype T cells, and reduced proliferation and cytokine production) are considered to be critical contributors to age‐associated‐immune dysfunction (Nikolich‐Zugich, 2008). From the standpoint of T‐cell senescence and intrinsic alterations of T‐cell signaling, it is important to study the roles of inhibitory molecules in aged T cells (Fulop *et al*., 2014). Recently, CD4^+^ and CD8^+^ T cells from aged mice have been shown to express several inhibitory receptor molecules, including PD‐1, LAG‐3, CTLA‐4, and KLRG1, all of which may be associated with defective T‐cell responses (Channappanavar *et al*., 2009; Shimada *et al*., 2009; Decman *et al*., 2012). It has also been suggested that a PD‐1 blockade may partially restore T‐cell function (Lages *et al*., 2010). Thus, understanding the increased T‐cell inhibitory signaling that occurs with aging is an important step toward developing approaches to rescue T‐cell senescence.

Prolonged expression of inhibitory molecules is a well‐known feature of T‐cell exhaustion in both human and mouse chronic infection models. For instance, in chronic lymphocytic choriomeningitis virus (LCMV)‐infected mice, PD‐1 is highly expressed on the surface of T cells compared to functional memory T cells, and these PD‐1^+^ CD8^+^ T cells fail to proliferate when stimulated, display defects in effector functions, and are called exhausted T cells (Barber *et al*., 2006). Exhausted T cells have also been identified in different viral infections, such as HIV and hepatitis A and B virus (Sharpe *et al*., 2007; Keir *et al*., 2008). Several previous studies of LCMV, HIV, and HCV infections have suggested that Tim‐3 is another inhibitory marker of exhausted T cells (Jones *et al*., 2008; Golden‐Mason *et al*., 2009; Jin *et al*.,^2010^). Tim‐3 was initially identified in IFN‐γ‐secreting Th1 cells and CD8^+^ T cytotoxic type 1 (Tc1) cells (Monney *et al*., 2002). By interacting with its ligand, Galectin‐9, Tim‐3 causes T cells to undergo cell death, which leads to peripheral tolerance (Rodriguez‐Manzanet *et al*., 2009). Tim‐3 is expressed on PD‐1^+^ CD8^+^ T cells, and co‐blockade of Tim‐3 and PD‐1 restores the functions of exhausted CD8^+^ T cells during LCMV infection (Jin *et al*., 2010). Furthermore, Tim‐3 is also known to be expressed on the surface of CD8^+^ tumor‐infiltrating lymphocytes in tumor‐bearing mice (Sakuishi *et al*., 2010), which suggests its potential as a therapeutic target in various diseases.

Here, we found that aging in mice is associated with increased expression of Tim‐3 on CD8^+^ T cells. These Tim‐3‐expressing CD8^+^ T cells in aged mice showed high levels of several inhibitory molecules and had proliferative defects following homeostatic or TCR stimulation, which are features of exhausted CD8^+^ T cells. Aged Tim‐3^+^PD‐1^+^ CD8^+^ T cells showed decreased production of inflammatory cytokines, such as TNF‐α, but increased production of IL‐10 compared to Tim‐3^−^PD‐1^+^ cells. Furthermore, these cells were able to induce normal CD8^+^ T cells to produce IL‐10. Our study suggests that this aging‐related Tim‐3 expression defines more severely exhausted CD8^+^ T cells, which could contribute to the environmental changes with aging during immunological events.

## Results

### Tim‐3‐ and PD‐1‐coexpressing CD8^+^ T cells accumulate in aged mice

**Figure 1:**
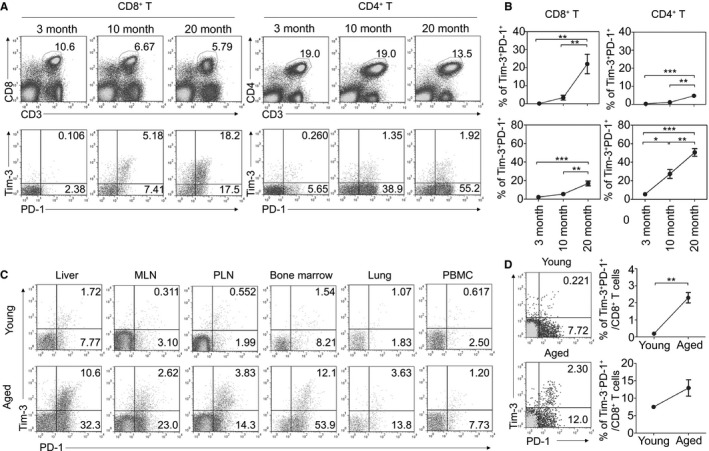
Tim‐3‐ and PD‐1‐coexpressing CD8^+^ T cells accumulate in aged mice. (A, B) The expression of Tim‐3 and PD‐1 in splenic CD8^+^ and CD4^+^ T cells was analyzed in naïve B6 mice aged 3, 10, and 20 months (n = 6 per group). Representative lymphocyte‐ and CD3^+^ CD8^+^‐gated FACS plots (A) and graphs of percentages of indicated subsets (B) are shown. (C) The expression of Tim‐3 and PD‐1 was analyzed in the indicated organs of young (2–3 months) and aged (19–22 months) mice. Representative CD3^+^ CD8^+^‐gated FACS plots are shown with percentages of Tim‐3^+^ PD‐1^+^ (upper right) and Tim‐3^−^ PD‐1^+^ (lower right) among CD8^+^ T cells. (D) The expression of Tim‐3 and PD‐1 on CD8^+^ T cells in the thymus of the young and aged mice. One‐way ANOVA with Bonferroni's post hoc (B) and unpaired two‐tailed *t‐*test (D). Error bars represent SEM. *P <* *0.05; **P <* *0.01; ***P <* *0.001. All data are representative of three independent experiments.

It has been proposed that impaired T‐cell function during aging is associated with an increase in the population of PD‐1‐expressing T cells (Channappanavar *et al*., 2009; Shimada *et al*., 2009; Lages *et al*., 2010; Decman *et al*., 2012). To address the relationship between the exhaustion of T cells and aging more specifically, we examined the expression of Tim‐3, which, along with PD‐1, defines T‐cell exhaustion during chronic infection or in tumors (Jin *et al*., 2010; Sakuishi *et al*., 2010) but has never been studied in a model of aging. To this end, we tested its expression on splenic T cells in naïve B6 aged mice at various ages: aged (20‐month‐old), mid‐aged (10‐month‐old), and young (3‐month‐old) mice. The results showed a dramatic increase in Tim‐3‐expressing cells, especially on CD8^+^ T cells, which accounted for approximately 20% of the total CD8^+^ T cells in aged mice (Fig. 1A). Tim‐3‐expressing cells were also detected in aged or mid‐aged CD4^+^ T cells, but the increase was not as remarkable as that in CD8^+^ T cells. Interestingly, most Tim‐3 expressing cells were shown to co‐express PD‐1, and the frequency of CD8^+^ T cells co‐expressing Tim‐3 and PD‐1 was increased approximately 20 times more than in young mice and 2–3 times more than in mid‐aged mice. On the other hand, over 50% of aged CD4^+^ T cells were PD‐1^+^, which is in agreement with previous reports, but most did not express Tim‐3 (Fig. 1A,B). These results indicate that aged T cells have different phenotypic characteristics that depend on the T‐cell subsets; aged CD8^+^ T cells include an approximately equal proportion of Tim‐3^+^PD‐1^+^ and Tim‐3^−^PD‐1^+^ cells, implicating the importance of Tim‐3 in CD8^+^ T cells compared to CD4^+^ T cells. We found that the increase of Tim‐3^+^PD‐1^+^ CD8^+^ T cells was also detected in aged Balb/c mice (Fig. S1). The considerable increase in Tim‐3^+^ cells was observed in most organs, and it was markedly elevated in liver and bone marrow, as it was in the spleen (Fig. 1C). Aged CD8^+^ T cells in the thymus also included more Tim‐3^+^ cells than young cells (Fig. 1D). Collectively, these data indicate that Tim‐3‐expressing cells accumulate in the aged CD8^+^ T‐cell population and that they co‐express PD‐1.

### Phenotypic characterization of Tim‐3‐expressing CD8^+^ T cells in aged mice

**Figure 2:**
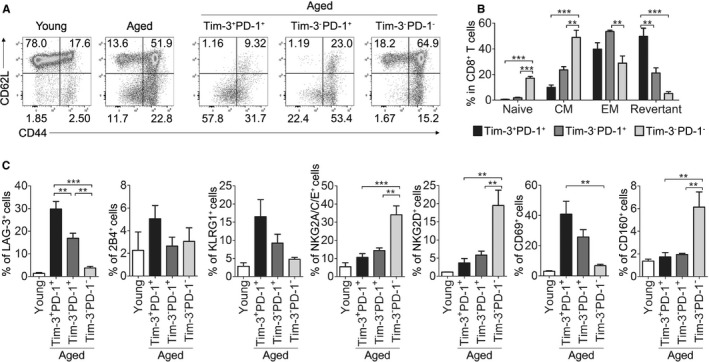
Phenotypic characterization of Tim‐3‐expressing CD8^+^ T cells in aged mice. (A, B) The expression of CD44 and CD62L in CD8^+^ T cells from aged and young mice (left), and in each aged CD8^+^ T‐cell subset (right) (n = 5). Representative CD3^+^ CD8^+^‐gated FACS plots are shown with percentages of each quadrant; CD44^lo^ CD62L^hi^ (Naïve), CD44^hi^ CD62L^hi^ (Central memory), CD44^hi^ CD62L^lo^ (Effector memory), and CD44^lo^ CD62L^lo^ (Revertant) (A). The statistical graph shown in (B). (C) The expression of various surface molecules on each aged CD8^+^ T‐cell subset was analyzed by flow cytometry. One‐way ANOVA with Bonferroni's post hoc (B, C). Error bars represent SEM. *P <* *0.05; **P <* *0.01; ***P <* *0.001. All data are representative of three independent experiments

To examine how the cells might be differentiated by their expression of Tim‐3 on PD‐1^+^ CD8^+^ T cells, we characterized the surface phenotype of aged CD8^+^ T‐cell subsets in Tim‐3^+^PD‐1^+^, Tim‐3^−^PD‐1^+^, and Tim‐3^−^PD‐1^−^ cells. Consistent with previous findings of effector/memory phenotypes, young CD8^+^ T cells in splenocytes contained a high proportion of naïve (CD44^lo^CD62L^hi^) cells and a low proportion of central memory (CM; CD44^hi^CD62L^hi^) cells, whereas aged CD8^+^ T cells contained a high proportion of CM and effector memory (EM; CD44^hi^CD62L^lo^) phenotype cells (Fig. 2A) (Ernst *et al*., 1993). Consistent with the previous study, most Tim‐3^−^PD‐1^+^ cells are of the memory phenotype (CD44^hi^) and that a large numbers of these cells are EM cells (Fig. 2A,B) (Lages *et al*., 2010). In contrast, Tim‐3^+^PD‐1^+^ CD8^+^ T cells are mostly CD62L^lo^ T cells, and the expression levels of CD44 in these cells are mainly distributed from intermediate to low, which characterizes them as revertant phenotype cells (Figs 2A,B, and S2) (Akbar & Fletcher, 2005; Lages *et al*., 2010). These data indicate that PD‐1‐expressing CD8^+^ T cells in aged mice exist in two different subsets that can be separated based on their Tim‐3 expression and that Tim‐3^+^PD‐1^+^ cells may represent a differentiation status that is distinct from that of Tim‐3^−^PD‐1^+^ cells. Notably, the effector/memory phenotype of Tim‐3^+^ cells in aging animals was similar to the phenotype of tumor‐infiltrating Tim‐3^+^ cells (Sakuishi *et al*., 2010).

To further characterize the exhausted phenotype of aged Tim‐3^+^PD‐1^+^ CD8^+^ T cells, we next examined the expression of several surface molecules that were shown to be up‐ or down‐regulated in exhausted CD8^+^ T cells in a chronic infectious mouse model (Blackburn *et al*., 2009). As a result, most of the exhaustion‐related molecules showed the same tendencies as those in the chronic infection model, and their expression levels were similar between aged Tim‐3^+^PD‐1^+^ and Tim‐3^−^PD‐1^+^ cells (Fig. 2C). Notably, the expression of LAG‐3 on Tim‐3^+^PD‐1^+^ cells was found to be significantly higher compared to Tim‐3^−^PD‐1^+^ cells. Of interest, the expression of CD160 was opposite to what was observed in chronic infection‐induced exhausted cells (Blackburn *et al*., 2009), which indicates that age‐related exhaustion is not identical to chronic infection‐induced exhaustion. Collectively, these results show that Tim‐3^+^PD‐1^+^ and Tim‐3^−^PD‐1^+^ CD8^+^ T cells in aged individuals display mostly similar exhausted phenotypes, but they are distinguishable from each other and from other types of exhausted cells based on some aspects of their surface molecule profiles.

### Aged Tim‐3^+^PD‐1^+^ CD8^+^ T cells fail to proliferate under TCR stimulation

**Figure 3:**
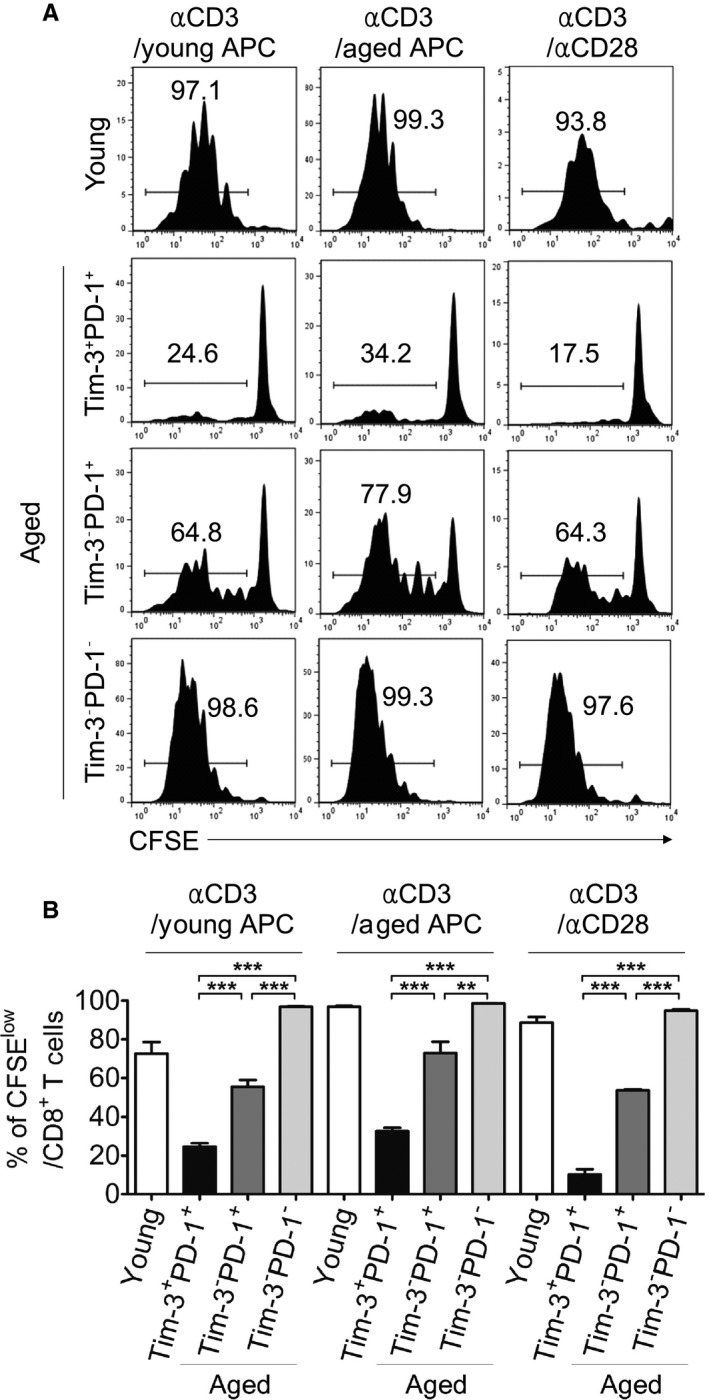
Aged Tim‐3^+^ PD‐1^+^ CD8^+^ T cells fail to proliferate under TCR stimulation. (A, B) The sorted CD8^+^ T‐cell subsets from three young and five aged mice were pooled and stimulated with anti‐CD3 Ab plus T‐cell‐depleted APCs of young or aged mice, or anti‐CD3 Ab plus anti‐CD28 Ab (n = 3). Three days later, the CFSE dilution was analyzed by flow cytometry. Representative FACS plots are shown in (A). Numbers indicate the percentages of cells that divided more than once. The statistical graph is shown in (B). One‐way ANOVA with Bonferroni's post hoc (B). Error bars represent SEM. *P <* *0.05; **P <* *0.01; ***P <* *0.001. All data are representative of two independent experiments.

Tim‐3‐ and PD‐1‐expressing CD8^+^ T cells display more severe exhaustion in chronic infection and tumor models than Tim‐3^−^PD‐1^+^ cells, as indicated by their failure to proliferate and produce effector cytokines (Jin *et al*., 2010; Sakuishi *et al*., 2010). To determine whether Tim‐3^+^PD‐1^+^ CD8^+^ T cells in aged mice display functionally exhausted properties, the proliferative capacities of aged CD8^+^ T cells in the presence of TCR stimulation were analyzed. Sorted cells representing each subset were cultured with stimulation by anti‐CD3/anti‐CD28 Ab or anti‐CD3 Ab along with young or aged T‐cell‐depleted antigen presenting cells (APCs). Compared to young CD8^+^ T cells or aged Tim‐3^−^PD‐1^−^ CD8^+^ T cells, which showed high levels of proliferation in all stimulation conditions (over 90%), the proliferative activity of Tim‐3^−^PD‐1^+^ and Tim‐3^+^PD‐1^+^ CD8^+^ T cells was markedly impaired. Interestingly, Tim‐3^+^PD‐1^+^ CD8^+^ T cells showed more reduced proliferation than Tim‐3^−^PD‐1^+^ cells (Fig. 3A,B). Thus, our results demonstrate the Tim‐3^+^PD‐1^+^ CD8^+^ T cells from aged mice are severely hyporesponsive to TCR stimulation, which supports the hypothesis that these cells are a more exhausted CD8^+^ T‐cell population than Tim‐3^−^PD‐1^+^ cells.

### Aged Tim‐3^+^PD‐1^+^ CD8^+^ T cells show impaired responses to homeostatic cytokine signals and lymphopenic *in vivo* conditions

**Figure 4:**
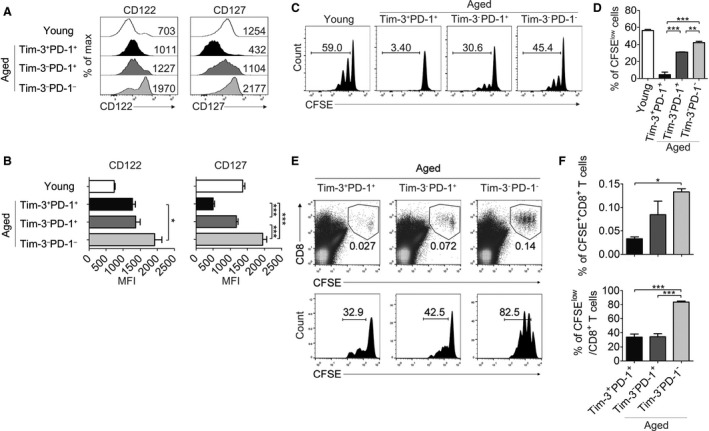
Aged Tim‐3^+^ PD‐1^+^ CD8^+^ T cells show impaired responses to homeostatic cytokine signals and lymphopenic *in vivo* conditions. (A, B) The expression of CD122 and CD127 in young or aged (n = 5) CD8^+^ T‐cell subsets was analyzed; representative histograms (A) and a statistical graph of gMFI (B) are shown (n = 5). (C, D) Sorted aged CD8^+^ T‐cell subsets and young CD8^+^ T cells were labeled with CFSE and cultured with IL‐7 and IL‐15 for 6 days (n = 3). Representative FACS plots of CFSE dilution (C) and the statistical graph of the percentage of CFSE ^low^ cells (D) are shown. Numbers indicate the percentage of cells that divided more than once. (E, F) Sorted aged CD8^+^ T‐cell subsets were labeled with CFSE and adoptively transferred into Rag‐1 KO recipient mice (n = 4 per group) which were sacrificed 5 days after transfer. CFSE ^+^ donor CD8^+^ T cells in the spleen and lymph nodes were analyzed. Representative FACS plots are shown in (E). Each number indicates the frequency among total lymphocytes (upper) and the percentage of cells that proliferated more than once (lower). Each graph is shown (F). One‐way ANOVA with Bonferroni's post hoc (B, D, F). Error bars represent SEM. *P <* *0.05; **P <* *0.01; ***P <* *0.001. All data are representative of three independent experiments.

Exhausted CD8^+^ T cells generated by chronic infection display low responsiveness to the homeostatic cytokines IL‐7 and IL‐15, and they fail to survive when adoptively transferred (Wherry, 2011). To identify this property in aged Tim‐3‐expressing CD8^+^ T cells, we first analyzed the expression of homeostatic cytokine receptors, including CD122 (IL‐15Rβ) and CD127 (IL‐7Rα), on each subset (Fig. 4A,B). Interestingly, aged Tim‐3^+^PD‐1^+^ CD8^+^ T cells expressed a comparable level of CD122 with Tim‐3^−^PD‐1^+^ cells but lower than Tim‐3^−^PD‐1^−^ cells; they expressed the lowest level of CD127. Next, we tested whether proliferation of Tim‐3‐expressing CD8^+^ T cells was also attenuated to IL‐7 and IL‐15 by culturing sorted Tim‐3^+^PD‐1^+^, Tim‐3^−^PD‐1^+^, or Tim‐3^−^PD‐1^−^ CD8^+^ T cells with IL‐7 and IL‐15 (Fig. 4C,D). The proliferative capacity of Tim‐3^+^PD‐1^+^ CD8^+^ T cells was markedly impaired compared with Tim‐3^−^PD‐1^+^ or Tim‐3^−^PD‐1^−^ cells, which correlated with IL‐7 receptor expression. We also assessed the proliferative capacity of each sorted subset in a lymphopenic environment where homeostatic proliferation normally occurs rapidly as a result of a relative excess of trophic cytokines. In a Rag‐1 deficient host, Tim‐3^+^PD‐1^+^ cells also showed limited proliferation. Notably, although population of Tim‐3^−^PD‐1^+^ cells tended to be higher than that of Tim‐3^+^PD‐1^+^ cells, their expansion was similar. This may be because there are other factors that may be able to induce a weak expansion on Tim‐3^+^PD‐1^+^ cells *in vivo* in addition to IL‐7 and IL‐15 (Fig. 4E,F). These data demonstrate that Tim‐3‐expressing CD8^+^ T cells have limited reactivity to tropic cytokines, which is also a property of exhaustion (Wherry, 2011). From this result, it can be surmised that homeostatic cytokines may play a limited role in the maintenance of Tim‐3‐expressing CD8^+^ T cells.

### Tim‐3^+^PD‐1^+^ CD8^+^ T cells in aged mice appear to be generated through antigen encounters, but not by specific infection

**Figure 5:**
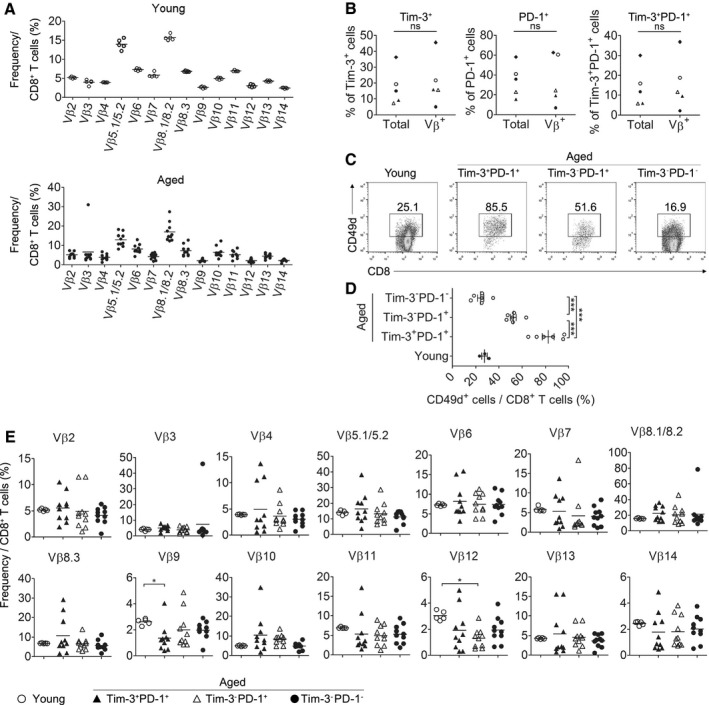
Tim‐3^+^ PD‐1^+^ CD8^+^ T cells in aged mice appear to be generated through antigen encounters but not by specific infection. (A) Splenic CD8^+^ T cells from young (upper, n = 5) and aged (lower, n = 10) mice were stained with a panel of TCR Vβ receptor antibodies, and the percentage of each Vβ^+^ cell is shown. (B) The graphs show the expression of Tim‐3 and PD‐1 on aged total CD8^+^ T cells and TCE Vβ^+^ cells defined as greater than the mean + 3 SDs of young Vβ use, ns = not significant. Each symbol indicates individual mice with TCE. (C, D) Young (n = 3) and aged (n = 6) CD8^+^ T‐cell subsets were stained for CD49d (integrin α4) and analyzed. Representative FACS plots (C) and graphs of the percentage of CD49d^+^ cells among indicated subsets (D) are shown, respectively. (E) The percentage of each Vβ^+^ cells in each aged CD8^+^ T‐cell subset was analyzed and shown as a graph. Paired *t‐*test (B). Kruskal–Wallis test (E). One‐way ANOVA with Bonferroni's post hoc (D). *P <* *0.05; **P <* *0.01; ***P <* *0.001. Data are representative of two or three independent experiments.

We next questioned how Tim‐3‐expressing CD8^+^ T cells develop and accumulate in naïve aged mice that are not manipulated by exogenous antigens. In aged individuals, CD8^+^ T cells undergo large clonal expansions of particular TCR Vβ repertoires (Clambey *et al*., 2007). In agreement with prior work, CD8^+^ T cells from some aged mice showed clonal T‐cell expansions (Fig. 5A) (Decman *et al*., 2012). To determine whether clonal expansion contributed to the process of accumulation of Tim‐3^+^PD‐1^+^ CD8^+^ T cells in aged mice, the expression of Tim‐3 on certain clonally expanded (TCE) Vβ‐carrying cells was compared with that on total CD8^+^ T cells from the same mice. TCE was defined as more than three SDs above the mean Vβ usage of the young CD8^+^ T cells (Decman *et al*., 2012). Following this criterion, five aged mice harbored TCE out of ten. The expression of Tim‐3 or PD‐1 on TCE Vβ^+^ cells was not significantly increased or decreased compared with that on total cells, suggesting that the induction of Tim‐3 expression is not specifically restricted to clonally expanded cells during aging but rather, that it occurs broadly in all CD8^+^ T cells (Fig. 5B).

Previous reports have shown that age‐associated clonal expansions shape two types of cells, based on their expression level of CD49d (α4‐integrin). One of these types is CD49d^hi^ T cells, the expansion of which is thought to be primed by exposure to antigens, and the other is the expansion of CD49d^lo^ cells, which is thought to be induced by cytokine‐dependent homeostatic proliferation (Clambey *et al*., 2008; Chiu *et al*., 2013). Compared to other aged CD8^+^ T‐cell subsets, Tim‐3^+^PD‐1^+^ CD8^+^ T cells showed the highest level of CD49d expression, indicating that they form as a result of the recognition of antigens rather than through cytokine‐dependent homeostatic proliferation (Fig. 5C,D). We next determined whether the results above were attributed to specific infections by assessing whether Tim‐3‐expressing CD8^+^ T cells have a dominant bias for the use of the Vβ chain recognizing a specific antigen. Although the Vβ9 chain of aged Tim‐3^+^PD‐1^+^ CD8^+^ T cells changed significantly, the decreased percentage of these repertoires represented only approximately 1–3%, while other Vβ repertoires of aged Tim‐3^+^PD‐1^+^ CD8^+^ T cells showed no significant difference compared with other aged CD8^+^ T‐cell subsets or young CD8^+^ T cells (Fig. 5E). These results indicate that the Tim‐3^+^PD‐1^+^ CD8^+^ T cells of aged mice do not form by interactions with specific antigens, such as infections, because the T‐cell repertoire of Ag‐specific CD8^+^ T cells from infectious mice narrow more severely as aging progresses (Bunztman *et al*., 2012). Collectively, these data suggest that Tim‐3^+^PD‐1^+^ CD8^+^ T cells in aged mice may be generated by exposure to a broad range of antigens over a lifespan, including self‐ or environmental antigens such as microbiota, rather than through specific infections.

### Aged Tim‐3^+^PD‐1^+^ CD8^+^ T cells display partially exhausted properties with increased production of IL‐10

**Figure 6:**
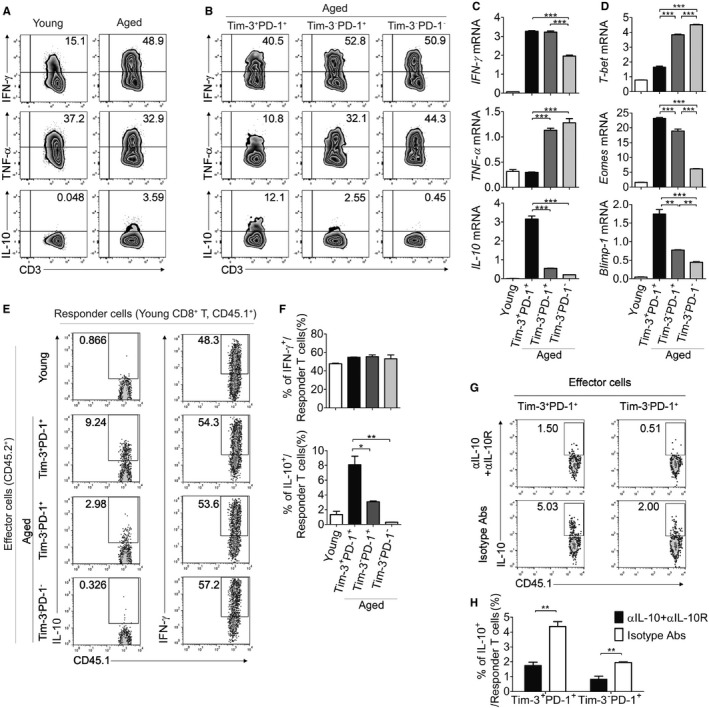
Aged Tim‐3^+^ PD‐1^+^ CD8^+^ T cells display partially exhausted properties with increased production of IL‐10. (A, B) Production levels of several cytokines including IFN‐γ, TNF‐α, and IL‐10 in young (n = 5) and aged (n = 5) CD8^+^ T cells (A) or aged CD8^+^ T‐cell subsets (B) after PMA/ionomycin stimulation for 3 h were analyzed by ICS. Numbers indicate the percentage of indicated cytokine‐positive cells. (C, D) The mRNA expression of IFN‐γ, TNF‐α, IL‐10, T‐bet, Eomes, and Blimp‐1 were assessed by real‐time PCR in the young (n = 5) CD8^+^ T cells and sorted aged (n = 5) CD8^+^ T‐cell subsets. (E–H) CD45.1^+^ CD8^+^ T cells were co‐cultured with young or sorted aged CD8^+^ T‐cell subsets (CD45.2^+^) with anti‐CD3 Ab plus anti‐CD28 Ab for 1 day, and ICS was performed for IFN‐γ and IL‐10 levels in the CD45.1^+^ CD8^+^ T cells after PMA/ionomycin stimulation (n = 3). Representative FACS plots are shown in (E), and statistical graphs are shown in (F). Anti‐IL‐10 Ab and anti‐IL‐10R Ab were added into the co‐culture of (E) to neutralize IL‐10; representative FACS plots and a statistical graph of the percentages of IL‐10^+^ cells in CD45.1^+^ responder cells are shown (G, H). One‐way ANOVA with Bonferroni's post hoc (C, D, F). Unpaired two‐tailed *t‐*test (H). Error bars represent SEM. *P <* *0.05; **P <* *0.01; ***P <* *0.001. Data are representative of two or three independent experiments.

As has previously been shown, Tim‐3‐ and PD‐1‐expressing exhausted CD8^+^ T cells in chronic viral infections and tumor microenvironments are dysfunctional, as indicated by their loss of IFN‐γ and TNF‐α (Barber *et al*., 2006; Jin *et al*., 2010; Sakuishi *et al*., 2010). To investigate whether Tim‐3^+^PD‐1^+^ CD8^+^ T cells from aged mice exhibited similar exhausted functions compared to those from chronically infected or tumor‐bearing mice, in terms of cytokine producing abilities, the protein (Figs 6A,B, and S3) and mRNA (Fig. 6C) expression levels of IFN‐γ and TNF‐α were measured in these mice. Interestingly, Tim‐3‐expressing CD8^+^ T cells retained an ability to produce IFN‐γ similar to other subsets upon stimulation of PMA and ionomycin, and even expressed more mRNA than Tim‐3^−^PD‐1^−^ CD8^+^ T cells, whereas their expression of TNF‐α was significantly reduced. In agreement with findings regarding exhausted T cells in chronic infection, we also found that aged PD‐1 expressing CD8^+^ T cells showed T‐bet^lo^ and Eomesodermin (Eomes)^hi^ expression (Paley *et al*., 2012) (Fig. 6D). Interestingly, Tim‐3^+^PD‐1^+^ cells express lower T‐bet and higher Eomes than Tim‐3^−^PD‐1^+^ cells. Given the hierarchy in loss of function during other chronic disease‐mediated exhaustion, aged Tim‐3‐expressing CD8^+^ T cells are partially exhausted and remain in a stage of intermediate dysfunction, displaying intact IFN‐γ expression (Wherry, 2011). However, Tim‐3 expressing cells showed reduced IFN‐γ production when stimulated with anti‐CD3 Ab, indicating that their IFN‐γ producing ability was restrained by TCR dysfunction (Fig. S4).

Tim‐3^+^PD‐1^+^ CD8^+^ T cells resulting from chronic LCMV infection have been shown to express IL‐10 (Jin *et al*., 2010), although its role remains unclear. We examined the expression level of IL‐10 in aged Tim‐3^+^PD‐1^+^ CD8^+^ T cells and observed that it is markedly up‐regulated compared to the other aged CD8^+^ T‐cell subsets and young CD8^+^ T cells, not only in mRNA expression but also in cytokine production (Figs 6A–C and S3). Furthermore, Tim‐3‐expressing cells were shown to express a high level of Blimp‐1, which regulates IL‐10 transcription (Fig. 6D). To identify the influence of Tim‐3^+^PD‐1^+^ CD8^+^ T cells on normal CD8^+^ T cells, each sorted aged CD8^+^ T‐cell subset (CD45.2^+^) was cocultured with congenic young CD8^+^ T cells (CD45.1^+^) in the presence of TCR stimulation. Although IL‐10 is a well‐known suppressor of T‐cell response, no suppressive effect was observed in any group (data not shown). Unexpectedly, responder T cells cultured with aged Tim‐3^+^PD‐1^+^ CD8^+^ T cells produced a high level of IL‐10 compared to cells cultured with other aged T‐cell subsets (Fig. 6E,F). This effect was reduced when IL‐10 signals were blocked by treatment with anti‐IL‐10 and anti‐IL‐10R Abs, indicating that the IL‐10 expressed by aged CD8^+^ T cells is critical to inducing normal CD8^+^ T cells to express IL‐10 (Fig. 6G,H). Overall, our data suggest that age‐associated Tim‐3^+^PD‐1^+^ CD8^+^ T cells produce high levels of IL‐10 and have potential to promote the IL‐10‐dependent expression of IL‐10 in normal CD8^+^ T cells and that these activities may contribute to the increased levels of IL‐10 in the aged immune system (Spencer *et al*., 1996; Alvarez‐Rodriguez *et al*., 2012).

## Discussion

Understanding how aging affects the major components of the immune system, such as T cells, is important for decreasing susceptibility to aging‐associated diseases. Here, we demonstrate that Tim‐3‐expressing CD8^+^ T cells accumulate with aging and show exhausted properties according to multiple criteria, including their proliferative capacity, inhibitory surface molecule profile, and cytokine production. Recent studies have provided evidence that the phenotypic and functional exhaustion of T cells is related to aging‐mediated immune dysfunction (Lages *et al*., 2010; Decman *et al*., 2012). In many chronic disease models, exhausted T cells have been shown to coexpress PD‐1 and Tim‐3, which have been investigated to improve immune responses through the blockade of negative regulatory signals transduced by PD‐1 and Tim‐3 (Sakuishi *et al*., 2011). Tim‐3 is also expressed on IFN‐γ expressing effector cells transiently during acute infections (Sakuishi *et al*., 2011). Recently, a high level of PD‐1 expression was identified in both aged CD4^+^ and CD8^+^ T cells, and these cells showed impaired proliferative capacity and cytokine production after TCR stimulation (Lages *et al*., 2010; Decman *et al*., 2012). In the current study, our data demonstrate that PD‐1‐expressing cells can be divided into two subset, Tim‐3 positive and Tim‐3 negative populations, particularly in CD8^+^ T cells. We present the functional and phenotypic properties related to exhaustion in age‐associated Tim‐3‐expressing CD8^+^ T cells by comparing these properties with those of the Tim‐3^−^PD‐1^+^ population or those previously defined in exhausted T cells.

It is interesting that Tim‐3‐expressing CD8^+^ T cells in aged mice show substantially different effector/memory phenotypes compared to Tim‐3^−^PD‐1^+^ cells. Based on the observation that they display low expression of CD44 and CD62L, many Tim‐3^+^ cells could be revertant cells, which are close to replicative senescence (Akbar & Fletcher, 2005), whereas the majority of Tim‐3^−^PD‐1^+^ cells are composed of memory phenotype cells, which display a high level of expression of CD44. These data suggest that these two subpopulations of PD‐1‐expressing CD8^+^ T cells might be in different stages of differentiation and that Tim‐3 may be an important marker that distinguishes them. Many Tim‐3‐expressing cells do not belong to the memory population, which expresses a high level of CD44. They are therefore likely to be overlooked in studies with the accumulated memory T‐cell pool with aging. In solid tumor models, Tim‐3‐expressing CD8^+^ T cells have the same effector/memory phenotype with those in aging, but in nonsolid tumor models they show primarily an effector phenotype, which means that the phenotype of exhausted T cells can be distinct, depending on the disease model. Moreover, age‐related exhaustion could have a similarity to that of solid tumors (Sakuishi *et al*., 2010; Zhou *et al*., 2011). Of note, in human elderly subjects, revertant cells also accumulate (Akbar & Fletcher, 2005), and their expression of Tim‐3 remains to be evaluated in future research. Because inhibitory receptors can be upregulated by activation and differentiation, functionality should be tested together to define exhaustion (Fuertes Marraco *et al*., 2015).

CD8^+^ T cells undergo a loss of function in a hierarchical fashion with the multiple stages of exhaustion in that they lose certain properties in a stepwise manner (Wherry, 2011). Specifically, IL‐2 production and proliferative activity are first attenuated and then TNF‐α production is impaired in the intermediate stage. In more severe exhaustion, impaired production of IFN‐γ is detected, and eventually the cells are eliminated. In many studies, Tim‐3 has been shown to be expressed on the most severely exhausted CD8^+^ T cells that display impaired IFN‐γ production (Jin *et al*., 2010; Sakuishi *et al*., 2010). It is interesting that in aged mice, Tim‐3‐expressing CD8^+^ T cells show some exhausted characteristics, such as reduced proliferation and dysfunctional TNF‐α production, but they are able to express a normal amount of IFN‐γ. Although they have a potential to produce IFN‐γ, that seems to be hindered by TCR dysfunction. Therefore, a thorough understanding of the cognate antigens of exhausted T cells encountered during aging will be required to clarify their cytokine profile following antigen‐specific stimulation and to determine the cytotoxicity of CD8^+^ T cells. Additionally, the direct inhibitory role of Tim‐3 molecule has been controversial in exhausted T cells, and its role and the underlying mechanism in aging must be further elucidated (Ferris *et al*., 2014).

The profile of transcription factors clearly represents the exhaustion status of Tim‐3^+^ aged CD8^+^ T cells as the highest Blimp‐1 and Eomes and the lowest T‐bet expression (Fig. 6D). Because the expression of Eomes has been found to increase in the terminal progeny of exhausted T cells, it seems that Tim‐3^+^PD‐1^+^ cells among aged CD8^+^ T cells are most similar to the terminal progeny (Paley *et al*., 2012). Eomes may function instead of T‐bet to express IFN‐γ in those cells; however, this must be elucidated in future studies.

IL‐10, a potent immunosuppressive agent, is known to inhibit T‐cell activation and function either directly or indirectly through regulation of APCs (Moore *et al*., 2001). Accumulated evidence has shown IL‐10 appears to have an undisputed inhibitory effect on CD4^+^ T cells, but IL‐10 can have the opposite effects on CD8^+^ T cells, depending on the model (Brooks *et al*., 2010; Mumm *et al*., 2011). For instance, IL‐10 inhibits the priming of CD8^+^ T cells but stimulates activated CD8^+^ T cells *in vivo* (Emmerich *et al*., 2012). Of note, the production of IL‐10 has been shown to be elevated in both human and mice with aging (Spencer *et al*., 1996; Alvarez‐Rodriguez *et al*., 2012), which is considered to involve aging‐related attenuated immune responses, and it has also been shown that aged CD44^hi^ CD8^+^ T cells express IL‐10 (Decman *et al*., 2012). Our observations suggest that the Tim‐3^+^PD‐1^+^ population is the main source of IL‐10 production in CD8^+^ T cells. In a chronic infection model, Tim‐3^+^PD‐1^+^ CD8^+^ T cells also produce IL‐10, but the role of this increased production has not been studied. It is predicted that IL‐10 might be involved in suppression of immune responses based on its general action (Jin *et al*., 2010). Given the observation that IL‐10 and STAT3 signaling have been suggested as critical factors that promote and sustain CD8^+^ effector and memory T cells, IL‐10 might be essential for maintaining the Tim‐3‐expressing T‐cell population by contributing to its self‐renewal via STAT3 signaling, which should be explored in future investigations (Lee *et al*., 2007; Cui *et al*., 2011). This finding might explain how the population of exhausted T cells is sustained for so long, even though they display impaired proliferative ability. Tim‐3‐expressing T cells have limited responsiveness to IL‐7 and IL‐15 stimulation, which maintains naïve and memory T cells, implying the existence of other survival factors, such as IL‐10. In addition, we observed that priming young CD8^+^ T cells with aged Tim‐3^+^PD‐1^+^ cells promoted the production of IL‐10 in an IL‐10‐dependent manner. IL‐10 is produced by fully activated CD8^+^ T cells, such as after IL‐12 priming, and it increases the long‐term memory population (Lee *et al*., 2007). Therefore, Tim‐3^+^PD‐1^+^ cells might be involved in the process of the abnormal accumulation of memory cells with aging through the production of IL‐10 around the site of other CD8^+^ T‐cell stimulation.

One of the interesting issues explored in this study is the determination of what causes the generation and accumulation of Tim‐3‐expressing CD8^+^ T cells in aged mice. The accumulation of memory phenotype cells in the aged T‐cell population has been thought to be induced by mainly antigenic stimulation (Nikolich‐Zugich, 2008). According to our observations, Tim‐3 expression on aged CD8^+^ T cells is highly correlated with CD49d expression, implying that they are primarily generated by antigen encounters. However, Tim‐3^−^PD‐1^+^ cells display moderate levels of expression, meaning they are a rather mixed population that can be generated by antigen‐dependent or antigen‐independent mechanisms. With aging, the virtual memory cells, defined by their low expression of CD49d, also accumulate (Chiu *et al*., 2013; Renkema *et al*., 2014). We found that the majority of virtual memory cells belong to the Tim‐3^−^PD‐1^−^ population. These findings suggest that Tim‐3 could be used as a marker to separate the populations generated by antigen encounters from virtual memory cells in aged T cells. The next question is that of which antigen might be involved in the generation of Tim‐3‐expressing CD8^+^ T cells in aged individuals. Given that Tim‐3‐expressing CD8^+^ T cells are usually detected in chronic infections, it is doubtful that aged mice have been exposed to unidentified infections throughout their life time. The aged mice used in this study did not show specific pathogen infection or any symptoms indicating infection. In addition, the population of Tim‐3 expressing cells is not biased to certain TCR Vβ chains, even though the variability was increased, compared to young cells. This suggests that a broad range of antigens, rather than a specific antigen, is involved in the accumulation of Tim‐3‐expressing CD8^+^ T cells. It has been shown that T‐cell exhaustion occurs when an antigen is present in excessive amounts and cannot be cleared completely in a timely manner. Furthermore, the specific antigen is required to maintain this population; in its absence, the exhausted cells continue through the process to deletion (Wherry, 2011). In the case of aging, due to the calibrated threshold of T‐cell responses and the altered antigen expression, self‐ or environmental antigens might be able to act as cognate antigens that participate in age‐induced T‐cell exhaustion.

In conclusion, we have identified a new CD8^+^ T‐cell population in aged mice that is distinguishable by its expression of Tim‐3. The data we present in this study demonstrate that Tim‐3 expression specifically defines T cells with age‐associated exhaustion. Our comprehensive comparison of each CD8^+^ T‐cell subset will help increase understanding of alterations in T‐cell populations that occur with aging and to establish strategies for improving treatments of dysfunctions of the immune system in the elderly.

## Experimental procedures

### Cell isolation and sorting

Murine spleen, thymus, lymph node, bone marrow, and lung were homogenized using a cell strainer, and red blood cells (RBC) were lysed using an RBC lysis buffer. Hepatic lymphocytes were isolated using Percoll (GE Healthcare, Little Chalfont, UK) as previously described (Lee *et al*., 2012). Peripheral blood mononuclear cells (PBMC) were isolated using Histopaque‐1077 (Sigma‐Aldrich, St. Louis, MO, USA) following the manufacturer's protocol. In several *in vitro* and *in vivo* experiments, splenic CD8^+^ T cells were enriched using anti‐CD8α^+^ magnetic beads and a MACS LS column (Miltenyi Biotec, Bergisch Gladbach, Germany), then sorted into three subsets, Tim‐3^+^PD‐1^+^, Tim‐3^−^PD‐1^+^, and Tim‐3^−^PD‐1^−^ by FACSAria II (BD Biosciences, San Jose, CA, USA). The sort purities were more than 95%. To prepare the T‐cell‐depleted antigen presenting cells (APCs), splenocytes were stained with biotinylated anti‐CD3 mAb (Biolegend, San Diego, CA, USA) and antibiotin magnetic beads (Miltenyi Biotec), after which an MACS LD column (Miltenyi Biotec) was used.

### Cell staining and flow cytometry

Information about the antibodies used for flow cytometry is listed in Table S1. For staining of the mouse TCR Vβ chains, a mouse Vβ TCR Screening Panel (BD Biosciences) was used. Splenocytes of young and aged mice were stained and incubated for 15 min at 4 °C using the manufacturer's recommended staining volume. For intracellular cytokine staining (ICS), the cells were stimulated with anti‐CD3/CD28 Abs or PMA (50 ng ml^−1^)/ionomycin (500 ng ml^−1^, Sigma‐Aldrich), and GolgiPlug (BD Biosciences) for 3 h, fixed and permeabilized using a Cytofix/Cytoperm kit (BD Biosciences) following the manufacturer's protocol. Samples were collected by FACSCalibur or FACSAria III (BD Biosciences), and acquired data were analyzed with flowjo software (Tree Star, Ashland, OR, USA).

### *In vitro* proliferation assay

The sorted cells described above were labeled with 5 μm carboxyfluorescein diacetate succinimidyl ester (CFSE; Invitrogen, Carlsbad, CA, USA) and incubated for 15 min at 37 °C. CFSE‐labeled T cells were cultured for 72 h with immobilized anti‐CD3 (5 μg ml^−1^) and soluble anti‐CD28 (1 μg ml^−1^, Biolegend) mAb or anti‐CD3 mAb with young or aged T‐cell‐depleted APCs. For the cytokine driven homeostatic proliferation assay, CFSE‐labeled sorted T cells were cultured for 6 days with rmIL‐7 (10 ng ml^−1^) and rmIL‐15 (100 ng ml^−1^; eBioscience, San Diego, CA, USA). The cells were analyzed after the dead cells were gated out using Fixable Viability Dye 780 (eBioscience).

### Adoptive transfer

CFSE‐labeled sorted aged cells (3 × 10^5^ per mouse) were transferred intravenously into 6‐week‐old B6.Rag1 KO recipient mice. Five days post‐transfer, spleen and lymph node lymphocytes from these recipient mice were isolated, and the CFSE dilution was analyzed.

### Quantitative real‐time PCR assay

Total RNA was isolated from sorted aged T‐cell subsets using TRIzol reagent (Invitrogen) and reverse‐transcribed to cDNA using the amfiRivert II cDNA Synthesis Master Mix (Gendepot, Barker, TX, USA). Quantitative real‐time PCR was performed with the LightCycler 1.5 instrument (Roche Diagnostics, Indianapolis, IN, USA) and the SYBR premix Ex Taq (Takara, Otsu, Japan). Primers were purchased from Cosmo Genetech (Seoul, Koera), and their sequences are shown in Table S2. The expression of each transcript was analyzed relative to the reference gene transcript (HPRT).

### Statistical analysis

All data were analyzed using graphpad prism 5.0 (GraphPad Software, La Jolla, CA, USA). Sample size was determined by the ‘resource equation’ method (Charan & Kantharia, 2013), and value E was kept between 10 and 20. *P*‐values were determined using paired or unpaired two‐tailed *t‐*test for comparing two groups. For comparing more than two groups, we performed one‐way ANOVA and post hoc Bonferroni test. To assess the Vβ distribution of aged mice, the Kruskal–Wallis test with Dunn's post hoc was performed. *P *<* *0.05 were considered to be significant.

## Funding

The research was funded by a grant from the Mid‐career Researcher Program (No. 2015R1A2A1A10055844) of the National Research Foundation(NRF), the Ministry of Science, CT and Future Planning and the National R&D Program for Cancer Control (No. 0720500) of the Ministry of Health and Welfare.

## Conflict of interest

The authors declare no conflict of interest.

## Author contributions

K.A.L., K.S.S., G.Y.K., Y.C.S., C.H.K., and E.A.B. performed the experiments and analyzed the data. I.K.K. participated in the critical review of the manuscript. K.A.L., K.S.S., and C.Y.K. designed experiments and wrote the manuscript.

## Supporting information


**Data S1** Experimental procedures
**Fig. S1** Tim‐3 expression in the CD8^+^ T cells of Balb/c mice.
**Fig. S2** CD44 expression of aged CD8^+^ T cells subpopulations.
**Fig. S3** Cytokine production capacity of aged CD8^+^ T cells subpopulations.
**Fig. S4** Reduced IFN‐γ expression of aged Tim‐3^+^PD‐1^+^ CD8 T cells upon stimulation with anti‐CD3 and CD28 antibodies.
**Table S1** Antibodies used for the flow cytometry and cell sorting
**Table S2** Primer sequences used for the real‐time PCR
