# Supplementary material for: Characterization of age‐associated exhausted CD8+ T cells defined by increased expression of Tim‐3 and PD‐1
Source: Aging Cell. 2016 Jan 10;15(2):291–300. doi: 10.1111/acel.12435 (PMC4783346; doi:10.1111/acel.12435)
Supplement: Supplementary file 1 — Data S1 Experimental procedures Fig. S1 Tim‐3 expression in the CD8+ T cells of Balb/c mice. Fig. S2 CD44 expression of aged CD8+ T cells subpopulations. Fig. S3 Cytokine production capacity of aged CD8+ T cells subpopulations. Fig. S4 Reduced IFN‐γ expression of aged Tim‐3+PD‐1+ CD8 T cells upon stimulation with anti‐CD3 and CD28 antibodies. Table S1 Antibodies used for the flow cytometry and cell sorting Table S2 Primer sequences used for the real‐time PCR [file ACEL-15-291-s001.docx]

**Supporting information**

**Experimental procedures**

*Mice*

C57BL/6(B6) mice aged 6 weeks and 9 and 18-20 months were purchased from the Korea Research Institute of Bioscience and Biotechnology (Daejeon, Korea). B6.Rag1 KO mice were purchased from Jackson Laboratories (Bar Harbor, ME, USA). BALB/c and B6 CD45.1^+^ congenic mice were purchased from Charles River Laboratories (Wilmington, MA, USA). All mice were maintained under specific pathogen-free conditions in the Animal Facility for Pharmaceutical Research at Seoul National University. Young mice (aged 6-12 weeks), mid-aged mice (aged 9 months), and aged mice (aged more than 19 months) were used. All experimental protocols were approved by the Institutional Animal Care and Use Committee (IACUC) of Seoul National University.

*In vitro co-culture assay*

Sorted CD8^+^ T cell subsets from the aged mice (CD45.2^+^) were cocultured with MACS-sorted CD8^+^ T cells from the young CD45.1^+^ congenic mice with anti-CD3 and anti-CD28 Abs for 1 day and then restimulated by PMA/ionomycin for 3 hrs for the ICS assay. To neutralize IL-10, anti-IL-10 Ab and anti-IL-10R Ab (10 μg/ml, Biolegend) were used in co-culture.


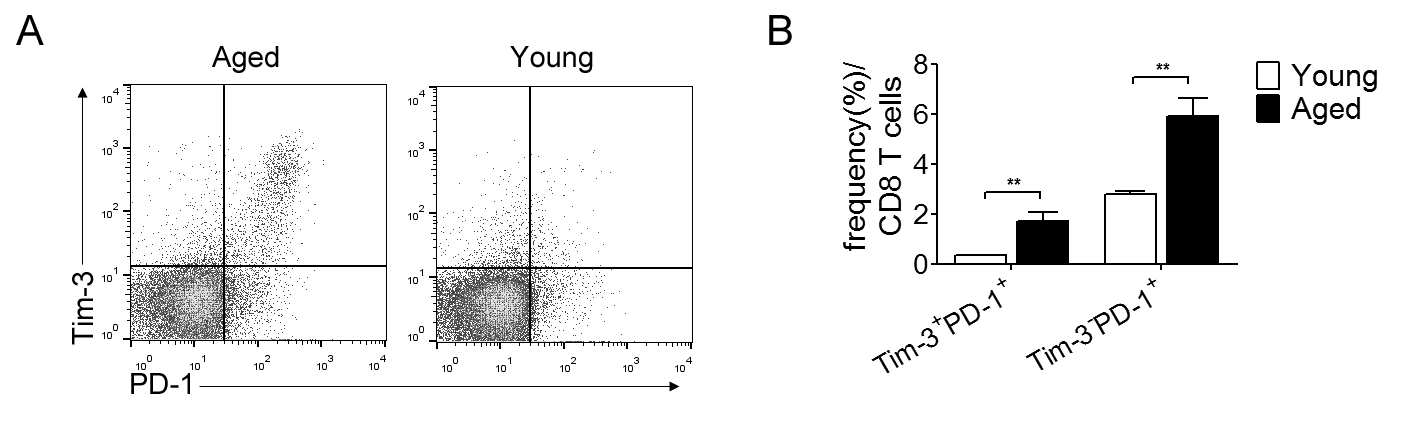


**Figure S1. Tim-3 expression in the CD8^+^ T cells of BALB/c mice.**

(A, B) The expression of Tim-3 and PD-1 was analyzed in splenocytes from young (n=3) and aged (n=5) BALB/c mice. Representative FACS plots of CD8^+^ T cells are shown in (A), and the statistical graph of percentage of each subset is shown in (B). Unpaired two-tailed *t* test was used for statistical analysis. Error bars represent SEM. ***p*<0.01. Data are representative of two independent experiments.


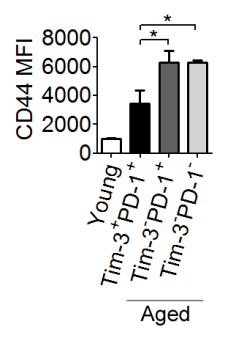


**Figure S2. CD44 expression of aged CD8^+^ T cell subpopulations**

The geometric mean fluorescence (gMFI) of CD44 was analyzed on aged Tim-3^+^PD-1^+^, Tim-3^-^PD-1^+^ and Tim-3^-^PD-1^-^ (n=5) and young (n=5) CD8^+^ T cells. One-way ANOVA with Bonferroni’s post-hoc was used for comparing three aged CD8^+^ T cell populations. Error bars represent SEM. **p*<0.05. Data are representative of three independent experiments.


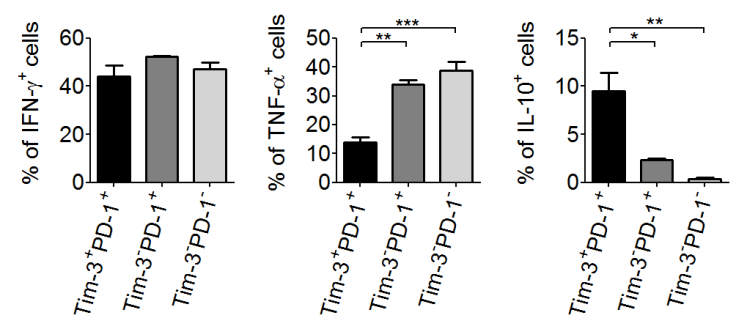


**Figure S3. Cytokine production capacity of aged CD8^+^ T cell subpopulations**

Aged (n=5) splenocytes were stimulated with PMA and ionomycin in the presence of Golgiplug for 3 hours. After stimulation, the levels of secretion of indicated cytokines in three aged CD8^+^ T cell subsets were measured by flow cytometry, and statistical graphs of the cytokine^+^ cell percentages are shown. One-way ANOVA with Bonferroni’s post-hoc was used for multiple comparisons. Error bars represent SEM. **p*<0.05; ***p*<0.01; ****p*<0.001. Data are representative of three independent experiments.


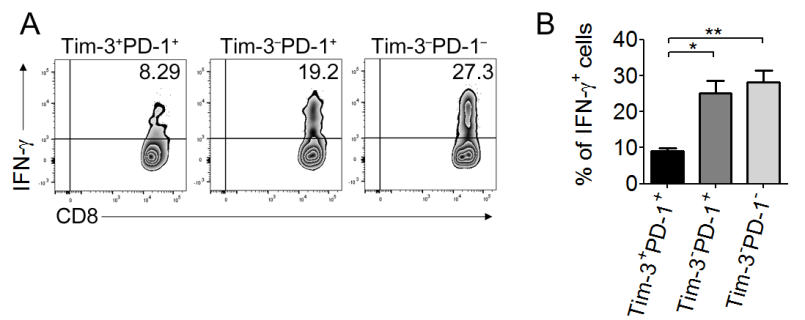


**Figure S4. Reduced IFN-γ expression of aged Tim-3^+^PD-1^+^ CD8 T cells upon stimulation with anti-CD3 and CD28 antibodies**

Aged (n=5) splenocytes were stimulated with anti-CD3 and CD28 antibodies in the presence of Golgiplug for 6 hours. After stimulation, the levels of IFN-γ production of three aged CD8 T cell subsets were measured by flow cytometry. Representative FACS plots (A) and a statistical graph of IFN-γ^+^ cell percentage (B) are shown. One-way ANOVA with Bonferroni’s post-hoc was used for multiple comparisons. Error bars represent SEM. **p*<0.05; ***p*<0.01. Data are representative of three independent experiments.

**Table S1. Antibodies used for the flow cytometry and cell sorting**

| **antibody** | **clone** | **fluorochrome** | **manufacturer** |
| --- | --- | --- | --- |
| 2B4 | M2B4(B6)458.1 | FITC | Biolegend |
| CD122 | TM-β1 | FITC | Biolegend |
| CD127 | SB/199 | FITC | Biolegend |
| CD160 | ebioCNX46-3 | Alexa Fluor®647 | ebioscience |
| CD3ε | 145-2C11 | PerCP/Cy5.5 | Biolegend |
| CD4 | RM4-5 | eFluor®450 | ebioscience |
| CD44 | IM7 | FITC | ebioscience |
| CD45.1 | A20 | PE/Cy7 | Biolegend |
| CD49d | R1-2 | FITC | Biolegend |
| CD62L | MEL-14 | APC | Biolegend |
| CD69 | H1.2F3 | APC | Biolegend |
| CD8α | 53-6.7 | APC | Biolegend |
|  |  | APC/Cy7 |  |
| Fixable viability dye 780 |  | APC-eFluor®780 | ebioscience |
| IFN-γ | XMG1.2 | APC | Biolegend |
|  |  | PE/Cy7 |  |
| IL-10 | JES5-16E3 | APC | Biolegend |
|  |  | PE/Cy7 |  |
| KLRG1 | 2F1/KLRG1 | APC | Biolegend |
| LAG-3 | C9B7W | Biotinylated | Biolegend |
| NKG2A/C/E | 20d5 | Biotinylated | ebioscience |
| NKG2D | C7 | Biotinylated | Biolegend |
| PD-1 | RMP1-30 | PE/Cy7 | Biolegend |
|  | J43 | FITC | ebioscience |
| Streptavidin |  | FITC | Biolegend |
| Tim-3 | RMT3-23 | PE | Biolegend |
| TNF-α | MP6-XT22 | APC | Biolegend |
|  |  | PE/Cy7 |  |

**Table S2. Primer sequences used for the real-time PCR**

| **Gene** | **Primer sequences** |
| --- | --- |
| IFN-γ | for : 5’–AACCCACAGGTCCAGCGCCA–3’ |
|  | rev : 5’–CACCCCGAATCAGCAGCGACT–3’ |
| TNF | for : 5’–GCCAGCCGATGGGTTGTACC–3’ |
|  | rev : 5’–CTTGGGGCAGGGGCTCTTGA–3’ |
| IL-10 | for : 5’–GCAGGGCCCTTTGCTATGGTG–3’ |
|  | rev : 5’–ATGAAGCGGCTGGGGGATGAC–3’ |
| Blimp-1 | for : 5’– ACT CAG TCG CAT TTG ATG GC –3’ |
|  | rev : 5’– GGT CAG TAA GGC TCT TGG GT –3’ |
| T-bet | for : 5’–ACAAGGGGGCTTCCAACAAT–3’ |
|  | rev : 5’–TGCGTTCTGGTAGGCAGTCA–3’ |
| Eomes | for : 5’– AGA ACC GTG CCA CAG ACC AA –3’ |
|  | rev : 5’– TCG TCA CAG GTT GCT GGA CA –3’ |
| HPRT | for : 5’– AAG ACT TGC TCG AGA TGT CAT GAA –3’ |
|  | rev : 5’– ATC CAG CAG GTC AGC AAA GAA –3’ |
